# The Physiology of Man, the Nervous System

**Published:** 1872-10

**Authors:** 


*The Physiology of Man; designed to represent the existing state of Physiological Science, as applied to the Human Body. Vol. IV. The Nervous System.* By Austin Flint, Jr., M. D. New York: D. Appleton & Co., 1872. Buffalo: Martin Taylor.

During the past few years no subject has received so much of experimental research and thoughtful attention as the nervous system. Our Medical Schools are realizing the importance of the subject, and chairs devoted to nervous affections are being established in many of our Colleges. The result of the inquiry which has thus been awakened is that much that in former years was included in the broad domain of the mythical and probable has now been taken to augment the slowly increasing collection of facts which are counted as known and positive.

The increased importance which this branch of science has lately assumed has rendered a text-book of this character an imperative necessity. Dr. Flint's work has been long expected, and its readers will not be disappointed in taking it as a guide in their studies concerning the arrangement and functions of the nervous system. The work is written in a clear intelligent manner, and but few facts that are not well established are included within its pages. The well known character of Prof. Flint as a Physiologist, and his reputation as a careful student will make this work one which will be sought after by many.

We heartily recommend it to our readers, not however, as a book with which to occupy an idle moment; its character demanding careful study and thoughtful reflection.

